# Australia: PISA Australia—Excellence and Equity?

**DOI:** 10.1007/978-3-030-59031-4_2

**Published:** 2020-11-24

**Authors:** Sue Thomson

**Affiliations:** Mathematics and Statistics, ISEG, University of Lisbon, Lisbon, Portugal; Australian Council for Educational Research, ACER, 19 Prospect Hill Rd, Camberwell, VIC 3124 Australia

## Abstract

Australia’s education system reflects its history of federalism. State and territory governments are responsible for administering education within their jurisdiction and across the sector comprising government (public), Catholic systemic and other independent schooling systems. They collaborate on education policy with the federal government. Over the past two decades the federal government has taken a greater role in funding across the education sector, and as a result of this involvement and the priorities of federal governments of the day, Australia now has one of the highest rates of non-government schooling in the OECD. Funding equity across the sectors has become a prominent issue. Concerns have been compounded by evidence of declining student performance since Australia’s initial participation in PISA in 2000, and the increasing gap between our high achievers and low achievers. This chapter explores Australia’s PISA 2018 results and what they reveal about the impact of socioeconomic level on student achievement. It also considers the role of school funding and the need to direct support to those schools that are attempting to educate the greater proportion of an increasingly diverse student population including students facing multiple layers of disadvantage.

## The Australian Education System and Goals for Education

Australia does not have a single national education system; its individual states and territories are responsible for their own education administration, although overall the structures are similar throughout the country. Policy collaboration between state and federal governments takes place in joint councils that include federal, state, and territorial government representatives. While most children attend government (or public) schools,^1^ approximately one-third attend non-government schools, in a sector comprising Catholic systemic schools and other independent schools.

State education departments recruit and appoint teachers to government schools, supply buildings, equipment, and materials, and provide limited discretionary funding for use by schools. In most jurisdictions, regional offices and schools have responsibility for administration and staffing, although the extent of responsibility varies across jurisdictions. Central authorities specify the curriculum and standards framework, but schools have autonomy in deciding curriculum details, textbooks, and teaching methodology, particularly at the primary and lower secondary levels. State authorities specify curriculum for Grades 11 and 12 and are responsible for examining and certifying final year student achievement for both government and non-government schools.

In the last two decades, in particular, the degree of involvement of the federal government and the degree of collaboration between state and territorial governments has increased. In 1989, the first declaration by joint federal and state education ministers arguing for nationally agreed goals of schooling national was released (the Hobart Declaration) (Australian Education Council 1989a, b). This was revised in 1999 and released as the Adelaide Declaration on National Goals for Schooling in the Twenty-First Century (Ministerial Council on Education, Employment, Training and Youth Affairs (MCEETYA) 1999). For the first time, one of the goals placed a value on equity: “Schooling should be socially just, so that: students’ outcomes from schooling are free from the effects of negative forms of discrimination based on sex, language, culture and ethnicity, religion or disability; and of differences arising from students’ socio-economic background or geographic location.”

In 2008, ministers of education agreed to the Melbourne Declaration on the Educational Goals for Young Australians (MCEETYA 2008), which outlined revised directions and aspirations for Australian schooling. The Melbourne Declaration elevated equity and excellence to the primary goal: “*Australian schooling promotes equity and excellence*”. In addition, it spelt out that “… all Australian governments and all school sectors must … ensure that the learning outcomes of Indigenous students improve to match those of other students …[and] ensure that socioeconomic disadvantage ceases to be a significant determinant of educational outcomes” (p. 7).

Since then, Australia’s national reform agenda has included the development of a national curriculum, and introduction of national standards for teachers and school leaders. Two national agencies—the Australian Curriculum, Assessment, and Reporting Authority (ACARA) and the Australian Institute for Teaching and School Leadership (AITSL)—were established to support these initiatives. The Australian Government’s National Assessment Program was established and includes PISA as one of several international assessments used as key performance measures for collecting data on the progress of Australian students toward the goals of the Melbourne Declaration. In 2013, the Australian Education Act was passed, which contained a broad range of national targets to ensure that Australia “provides a high quality and highly equitable system for all students”, and “for Australia to be placed, by 2025, in the top 5 highest performing countries based on the performance of school students in reading, mathematics and science” (Australian Government 2013, p. 3).

In the week following the release of the PISA 2018 results, serendipitously, the federal and state education ministers met in Alice Springs, in the Northern Territory, to discuss and agree on a revised statement of national goals. This new statement, the Alice Springs (Mparntwe) Education Declaration has, again, as its primary goal, “The Australian education system promotes excellence and equity”, and commits that “… the education community works to ‘close the gap’ for young Aboriginal and Torres Strait Islander students” (p. 16) and “governments and the education community must improve outcomes for educationally disadvantaged young Australians … such as those from low socioeconomic backgrounds, those from regional, rural and remote areas …” (Council of Australian Governments Education Council 2019, p. 17).

## Funding

To fully explain the methods and history of funding education in Australian schools would require a chapter on its own. In most OECD countries, non-government schools get little or no money from government funding—they are, after all, privately owned and operated. In Australia, the story is convoluted and complicated, and goes back to our origins as a British penal colony, with a population of largely Protestant English and Catholic Irish.^2^ As early as the 1830s, Governor Bourke tried to introduce schools modelled on the Irish National System, with students from all denominations educated in the one school. However, given the sectarianism of the time, these failed. Decades of division between church schools and government-managed schools ensued, and between the 1870s and 1890s each of the Australian colonies passed Education Acts that mandated that education be ‘free, compulsory, and secular’. This essentially stopped most financial assistance to church schools and made education a state responsibility. In addition to cutting them off from state funding, these Acts also cut Catholic and Protestant private schools loose from any state-imposed restrictions. The Protestant schools that remained separate at this time were largely the more elite high-fee schools.

The next episode relevant to the growth of the three systems in Australia occurred in the 1960s, when governments began giving money to church schools, with very few conditions. This is summed up perfectly by Bonnor and Caro (2007):

It is a fascinating study of good intentions, short-term solutions, political ambition and expediency, and the final death throes of the old Protestant versus Catholic prejudices that so bedeviled Australian society until the 1960’s (p. 35).

The post-war baby boom put huge strain on both government and Catholic schools, the latter of which had traditionally educated children from working class families and were the poor relations of the education system at the time. Fewer people were choosing a life in the church, and, for the first time, Catholic schools were having to employ (and pay) large numbers of lay teachers. In contrast, Protestant schools, also having to employ teachers, took a different path and resorted to charging higher fees, thereby limiting the access to wealthy families. State governments put pressure on the federal government for help in funding education, and eventually this started to occur in various forms. However, Bonnor and Caro (2007) point out that “Among the politics of the day one thing was entirely ignored: that along with public funding should go an established set of public obligations” (p. 37).

This approach to funding, put in place in the 1970s, has had ongoing repercussions that have never been reconciled in terms of funding for the three school sectors, with the funding agreement for Catholic schools flowing on to the rest of the non-Government sector. These repercussions include a change in perceptions of the role of the government schooling system. Connors and McMorrow (2010) noted that “at the beginning of significant Commonwealth funding of schools, the primary obligation of governments was to maintain government school systems at the highest standards, open to all, without fees or religious tests. In 1974, those obligations were enshrined in relevant Commonwealth legislation, but by 2011 they had been expunged from the legislation” (p. 32). By the last year of his government in 2007, Prime Minister John Howard had downgraded the level of education to be acquired from government schools to “… the safety net and guarantor of a reasonable quality education in this country” (Armitage 2007). While the Catholic system, in particular, had traditionally educated children from poor families, this is no longer the case, with many families choosing to send their children to these schools for aspirational, rather than religious reasons.^3^ The failure of successive governments to tie funding to obligations has provided subsidised private schools with a substantial advantage over their public counterparts, an advantage which is not mirrored in school systems in other countries. Bonnor and Caro (2007) conclude that:

The irony has been that the subsidies [to non-government schools], which were initially aimed at bringing poorly resourced private schools up to the resource and achievement levels of public schools, have continued unchecked until they have neatly reversed the original situation they were set up to rectify… public schools are now the resource-poor relations in the education system (p. 38).

In most other countries, there are only relatively small proportions of students attending non-government schools. While there has been a move away from government schools to the non-government sector over the past 20 years, there have been some returns over the past few years to government schools, however, currently, the Catholic system enrolls 23% of Australian secondary school children, independent schools 18% and government schools 59%. This is one of the highest rates of non-government schooling in the OECD.

Constitutionally, school education is the responsibility of the states, and they provide most of the funding for government schools (about 88% nationally). While it does not operate any schools itself, and is under no obligation to do so, the federal government provides the balance of funding to government schools and the majority of funding to non-government schools. According to the latest figures available on the website for the Department of Education, Skills and Employment (Australian Government 2020), around three-quarters of the funding for Catholic schools and less than one-half of the funding for independent schools is from public purses, compared to 95% of funding for government schools. Federal government funding is allocated based on an estimate of how much government funding each school requires to meet the educational needs of its students. This estimate is calculated by reference to the Schooling Resource Standard (SRS), which provides a base amount for every primary and secondary student, along with six loadings that provide extra funding for disadvantaged students and schools. For most non-government schools, the base amount is discounted or reduced by the anticipated capacity of the school community to financially contribute towards the school’s operating costs. This is called the ‘capacity to contribute’ assessment and it is based on a direct measure of median income of parents and guardians of the students at the school. This money is then provided to the state and territory governments and to organisations such as the Catholic education system—which then distribute the money to individual schools according to their own formulas, and with no requirement for transparency as to how funds are distributed.

**Fig. 1:**
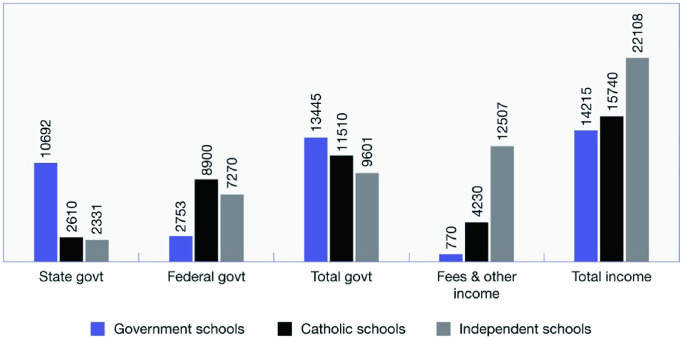
Australian school income by source per student, by school sector, 2017 (*Source* ACARA, National Report on Schooling data portal)

With widespread dissatisfaction among educational stakeholders in the equity of the funding system, 2011 saw a major review led by David Gonski. The primary aim of this review was to “develop a funding system for Australian schooling which is transparent, fair, financially sustainable and effective in promoting excellent outcomes for all Australian students” (Gonski 2011, p. xiii). Harking back to the aims of the early education agreements, the review argued that funding should aim to ensure that differences in educational outcomes were not the result of non-school factors such as a student’s socioeconomic background. One of the primary recommendations of the review panel was that “a significant increase in funding is required across all schooling sectors, with the largest part of this increase flowing to the government sector due to the significant numbers and greater concentration of disadvantaged students attending government schools. Funding arrangements for government and non-government schools must be better balanced to reflect the joint contribution of both levels of government in funding all schooling sectors” (Gonski 2011, p. xv). Unfortunately the Labor government of the time failed to implement the changes as directly recommended by the Gonski panel (making the promise that “no school will lose money”), and subsequent governments have made a variety of modifications to the model which, it is argued, have not delivered the funding system nor the benefits envisaged by Gonski (Bonnor & Shepherd 2016; Boston 2016; Goss & Sonnemann 2016; Rorris 2016). Over the past 10 years, funding increases have been misdirected towards private schools rather than to government schools. Data released by ACARA show that between 2009 and 2017, government funding (adjusted for inflation) for government schools was cut by $17 per student (−0.2%) while funding for Catholic schools increased by $1,420 per student (18.4%) and for independent schools by $1,318 (20.9%) (Cobbold 2019). To cap it off, while all schools are theoretically able to charge fees, such fees are not compulsory in government schools and are not able to be levied to the extent they are in non-government schools. While many government schools struggle with outdated and worn out facilities, lack of physical resources such as photocopy paper, broken down or inadequate toilet facilities and a lack of teaching staff, some elite independent schools are spending astonishing amounts of money on capital works, including theatres with orchestra pits, indoor Olympic size swimming pools, wellness centres, and equestrian centres. It is estimated that Australia’s four richest schools spent more on new facilities than the poorest 1,800 schools combined between 2013 and 2017 (Ting, Palmer & Scott 2019). The average funding per student, by school sector from all sources, for 2017, is shown in Fig. 1.

Curiously, however, government schools are funded at 85–90% of the Schooling Resource Standard (SRS), while Catholic and independent schools are currently funded at levels either close to 100% of their SRS or at levels even higher than this.

## Is Australia Meeting Its Goals for Schooling?

Given the importance attached to equity and excellence in the Australian National Education Goals since 1999, as well as the attempts to change the funding structures to try and ensure equitable outcomes for all students, it would seem timely to pause and review Australia’s progress towards attaining these goals, using the most recent release of PISA data in 2019.

### Is Australia Attaining Excellence?

Australia’s 2018 PISA results were met on their release in late 2019 with widespread shock and hand-wringing, even though scores have actually been declining since Australia’s initial participation in PISA in 2000. The most recent results saw Australia’s average scores drop to equal the OECD average in mathematical literacy, and those in reading and scientific literacy significantly lower than a decade ago, although still significantly higher than the OECD average.

**Fig. 2:**
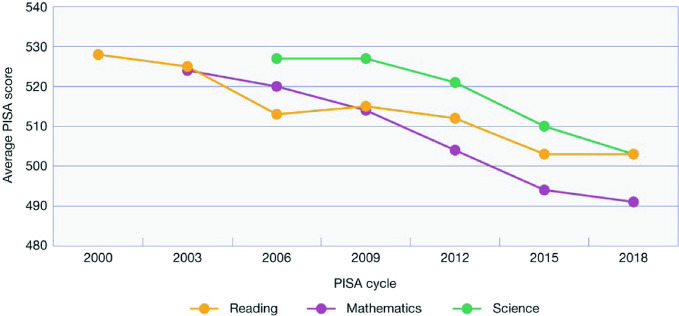
Australian students’ performance in PISA 2000–2018 (*Source* OECD 2019)

Figure 2 shows the average scores in achievement for reading, mathematical and scientific literacy for Australian students from 2000 to 2018. In 2000, when reading literacy was first assessed, Australian students achieved a mean score of 528 points, substantially as well as significantly higher than the OECD average of 500 points. In 2009, when it was again a major domain, the score had declined to 515 points, and then in 2018 to 503 points. This decline of 26 points represents a decline of about ¾ of a school year in terms of what students can do.^4^

In 2003, Australia’s average score in mathematical literacy was 524 points, again substantially as well as significantly higher than the OECD average of 500 points. In 2012 when mathematical literacy was again a major domain, the average score for Australian students was just 504 points, and in 2018 had declined further to 491 points. This score was not significantly different to the OECD average— which had also declined over time to 489 points—and represents a decline from 2003 of almost 1¼ years of schooling in what students can do.

In 2006, when scientific literacy was first assessed, Australia achieved a mean score of 527 points. In 2015, when it was again a major domain, the score had declined to 510 points, and in 2018 to 503 points. This represents almost one full school year decline between 2006 and 2018.

In 2011, the Gonski panel warned that:

Australian schooling needs to lift the performance of students at all levels of achievement, particularly the lowest performers. Australia must also improve its international standing by arresting the decline that has been witnessed over the past decade. For Australian students to take their rightful place in a globalised world, socially, culturally and economically, they will need to have levels of education that equip them for this opportunity and challenge (Gonski 2011, p. 22).

**Fig. 3:**
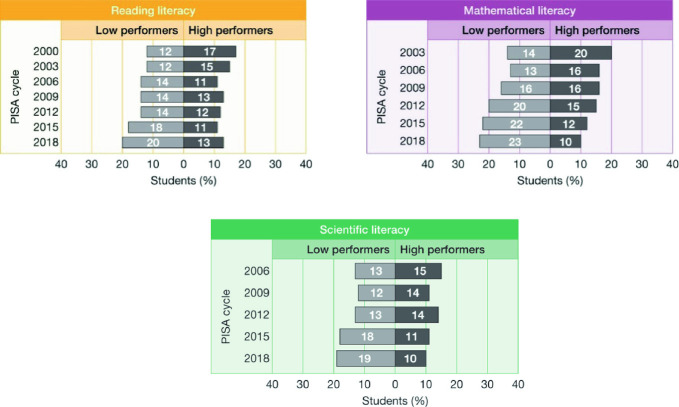
Percentages of high and low performers in reading, mathematical and scientific literacy, 2000–2018, Australia (*Source* OECD 2019)

Evidence suggests that this has not been the case. In Fig. 3, PISA proficiency levels in reading, mathematical and scientific literacy have been grouped into *high performers*, those who achieve at proficiency level 5 and above, and *low performers*, those achieving below proficiency level 2. In 2000, 17% of Australian students were high performers in reading literacy, and 12% low performers. In 2018, 13% were high performers in reading literacy and 20% low performers. In mathematical literacy the situation has become even more dire. In 2003, 20% of Australian students were high performers, 14% low performers. In 2018, 10% were high performers, and 23% low performers. In scientific literacy the situation has also deteriorated: from 13% low performers and 15% high performers in 2006 when scientific literacy was first assessed, to 19% low performers and 10% high performers in 2018.

**Fig. 4:**
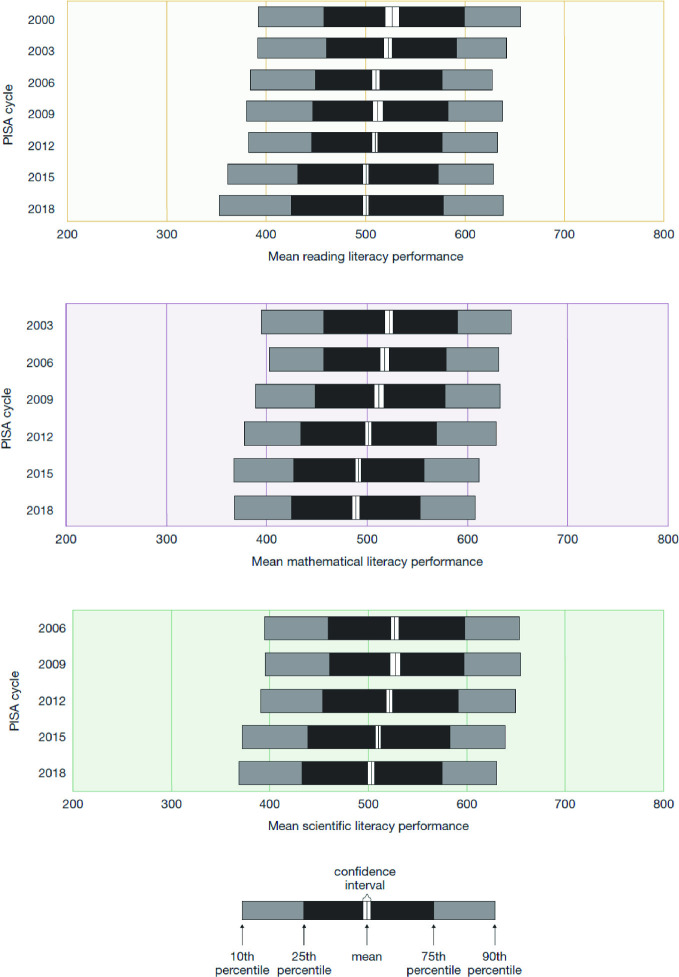
Distribution of student performance on the reading, mathematics and science literacy scales, PISA 2000–2018, Australia (*Source* Thomson et al. 2019)

Over time, the gap between the high achievers and the low achievers has increased, particularly in reading literacy. This is largely due to a larger decline at the lower percentiles of performance (Fig. 4). Over the PISA cycles, performance in reading literacy at the 10th percentile declined by 38 points (about 1.2 years of schooling), performance at the 90th percentile declined by 15 points (less than half a year of schooling). The difference between the highest and lowest percentiles in 2000 was 261 points (almost 8 years of schooling), which had increased to 284 points (8.6 years of schooling) in 2018. In mathematical literacy scores at the 10th percentile declined by 27 points (about one school year), and at the 90th percentile by 35 points (about 1¼ school years), between 2003 and 2018. The gap between highest and lowest remained roughly the same—246 score points in 2003 and 238 in 2018. Changes in scientific literacy scores have been similar: performance at the 10th percentile declined by 25 points (almost one year of schooling) between 2006 and 2018, and at the 90th percentile by a similar 23 points. In PISA 2006 the difference between high and low performers was 259 points and in 2018 it was 262 points.

### Is Australia Attaining Equity?

The Alice Springs (Mparntwe) Education agreement argues that “the education community must improve outcomes for educationally disadvantaged young Australians” (COAG Education Council 2019, p. 17), and identifies educationally disadvantaged as students from low socioeconomic backgrounds, Aboriginal and Torres Strait Islander students, and students from regional, rural, and remote areas—among others—but this chapter will concentrate on these groups.

#### Students from Low Socioeconomic Backgrounds

If a student’s social background is not a determinant of their achievement, then achievement levels would be evenly distributed across socioeconomic groups. To what extent is this the case for Australia?

The primary measure used by the OECD to represent socioeconomic background in PISA is the index of economic, social and cultural status (ESCS), which was created to capture the wider aspects of a student’s family and home background. The ESCS is based on three indices: the highest level of the father’s and mother’s occupations (known as the highest international social and economic index—HISEI), which is coded in accordance with the International Labour Organization’s International Standard Classification of Occupations; the highest educational level of parents in years of education (PARED); and home possessions (HOMEPOS). The index HOMEPOS comprises all items on the indices of family wealth (WEALTH), cultural resources (CULTPOSS), and access to home educational and cultural resources and books in the home (HEDRES). It must be noted that there have been some adjustments to the computation of ESCS over the PISA cycles.

**Fig. 5:**
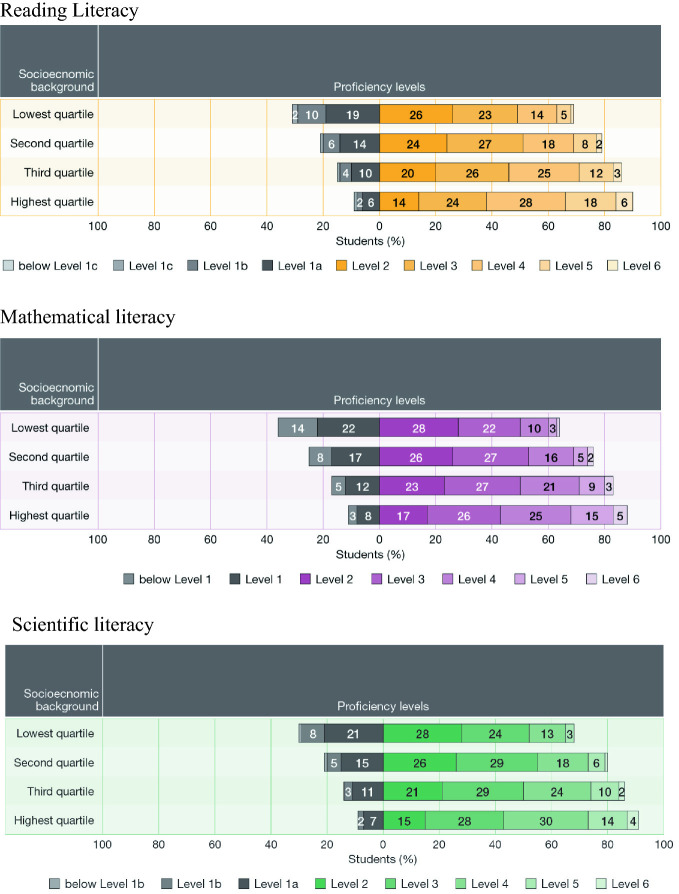
Percentages of students across the reading, mathematical and scientific literacy proficiency scales by socioeconomic background, PISA 2018, Australia (*Source* Thomson et al. 2019)

The average score for students who were in the lowest quartile of ESCS in PISA 2018 (disadvantaged students) was 460 points in reading literacy, compared to 549 points which was the average for those in the highest quartile (advantaged students). This difference of 89 points represents about 2.7 years of schooling. In mathematical literacy the average score for disadvantaged students was 451 points and for advantaged students 532 points, a difference of 81 points representing 2.9 years of schooling. In terms of international positions, these scores would place advantaged students at the same achievement level in reading literacy as those in the highest achieving PISA countries, B-S-J-Z China^5^ and Singapore, and disadvantaged students around the same level as the Slovak Republic and Greece. Figure 5 shows the distribution of proficiency levels in reading, mathematical and scientific literacy across socioeconomic background. Clearly, there *are* substantial differences in achievement across socioeconomic level in Australia in these key areas of literacy.

**Fig. 6:**
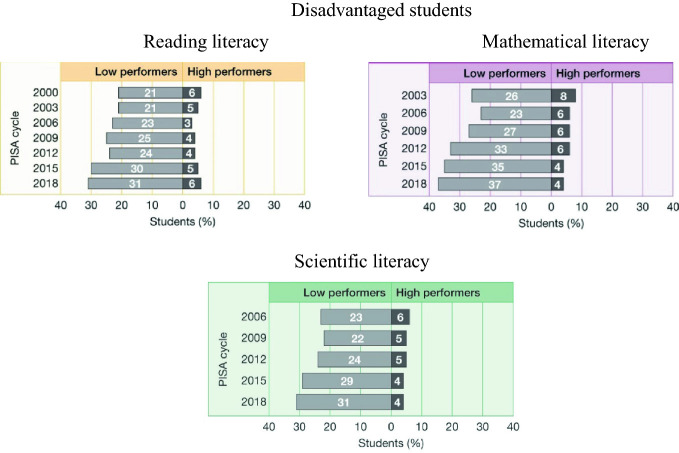
Proportions of low performers in reading, mathematical and scientific literacy for students from a low socioeconomic background over time, PISA 2000–2018, Australia (*Source* Thomson et al. 2019)

Moreover, this has been the case since the first administrations of PISA. In 2000, as shown in the top left panel of Fig. 6, 21% of disadvantaged students were low achievers in reading literacy. Results from the latest round of PISA in 2018 show that this situation has deteriorated, with 31% of disadvantaged students now classed as low performers. In 2003, 26% of disadvantaged students were low performers in mathematical literacy, and in 2018 this had risen to 37% of this group of students. In 2006, 23% of disadvantaged Australian students were low performers in scientific literacy, in 2018 this had risen to 31% of disadvantaged students.

**Fig. 7:**
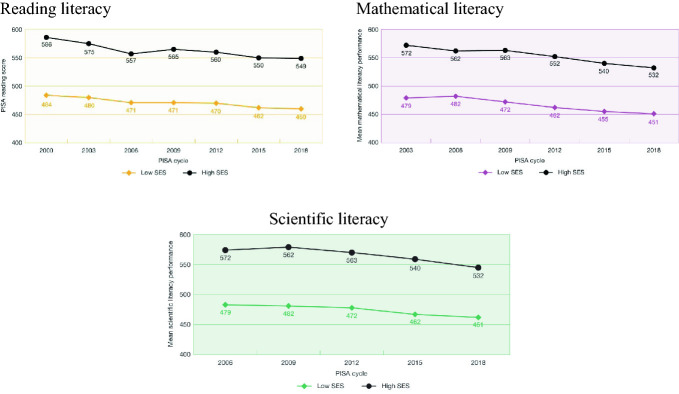
PISA reading, mathematical and scientific literacy scores over time, advantaged and disadvantaged students, PISA 2000–2018, Australia (*Source* Thomson et al. 2019)

What should be positive news is that the gap between the average score of advantaged and disadvantaged students has narrowed slightly in all three literacy areas (Fig. 7) from 102 point to 89 points in reading, from 92 points to 81 points in mathematical literacy, and from 91 points to 83 points in scientific literacy. It should be noted though that the gap only narrows from about 3 years of schooling to 2.7 years of schooling in reading literacy, from 3.3 years to 2.9 years in mathematical literacy, and from 3.4 years to 3.1 years in scientific literacy.

However, in reality, this narrowing is due to the larger decline in the scores of the advantaged students in all areas. In reading literacy, the average scores for disadvantaged students declined by 24 points—where the decline for those in the highest quartile was 37 points. In mathematical literacy the average score for disadvantaged students declined by 28 points, while the decline for advantaged students was 40 points. In scientific literacy the average score for disadvantaged students declined by 21 points and the decline for advantaged students 29 points.

#### Students from an Aboriginal or Torres Strait Islander Background

Traditionally, students from an Aboriginal and Torres Strait Island background have been poorly served by the Australian education system (Gray & Beresford 2008). Reflecting on the first of the reports on *Overcoming Indigenous Disadvantage* in 2003, the Steering Committee Chair commented that “It is distressingly apparent that many years of policy effort have not delivered desired outcomes: indeed in some important respects the circumstances of Indigenous people appear to have deteriorated or regressed” (Steering Committee for the Review of Government Service Provision 2005, p. xix).

**Fig. 8:**
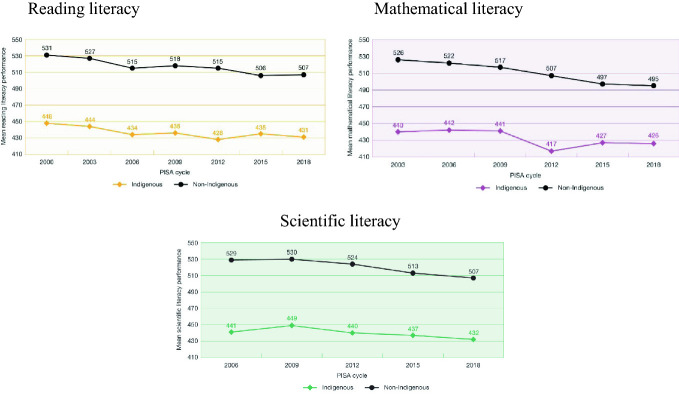
Mean reading, mathematical and scientific literacy scores over time, for Indigenous and non-Indigenous students, PISA 2000–2018, Australia (*Source* Thomson et al. 2019)

PISA 2000 provided a first measure of the gap between Indigenous and non-Indigenous students, with a gap of 83 points in reading literacy (2.5 years of schooling) followed by similar gaps in subsequent rounds of PISA - 86 points in mathematical literacy in 2003 (3.1 years of schooling) and 88 points in scientific literacy in 2006 (3.3 years of schooling). In PISA 2018, a decline in the scores of non-Indigenous students in all three assessment areas brought the gaps to 76 points (2.3 years of schooling) in reading literacy, 69 points (2.5 years of schooling) in mathematical literacy and 75 points (2.8 years of schooling) in scientific literacy. Again, not the envisaged means of closing the gap (Fig. 8).

**Fig. 9:**
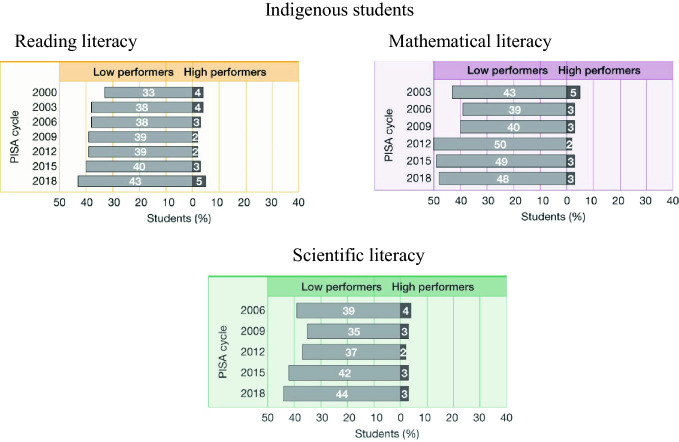
Proportions of low performers in reading, mathematical and scientific literacy for Indigenous students over time, PISA 2000–2018, Australia (*Source* Thomson et al. 2019)

Of particular concern is the proportion of low-performing Indigenous students in all three assessment areas, and this has worsened over time (Fig. 9). In 2000, 33% of Indigenous students were low performers in reading literacy, and in 2018 this had increased to 43 per cent. In mathematical literacy in 2003, 43% of Indigenous students were low performers, and this has hovered around the 50% mark in recent years. In scientific literacy, 39% of Indigenous students were low performers, and this increased to around 44% in 2018. These proportions are also most likely an underestimate of the actual proportions as PISA is unable to assess many Indigenous students—those who live in extremely remote areas, those who do not have instruction in English, and those who do not attend on the days of testing.

#### Students from Regional and Remote Areas

In Australia in 2018, participating schools were coded broadly as:

- metropolitan—mainland capital cities or major urban districts with a population of 100,000 or more
- provincial—provincial cities and other non-remote provincial areas
- remote—areas with very restricted or very little accessibility to goods, services and opportunities for social interaction.

**Fig. 10:**
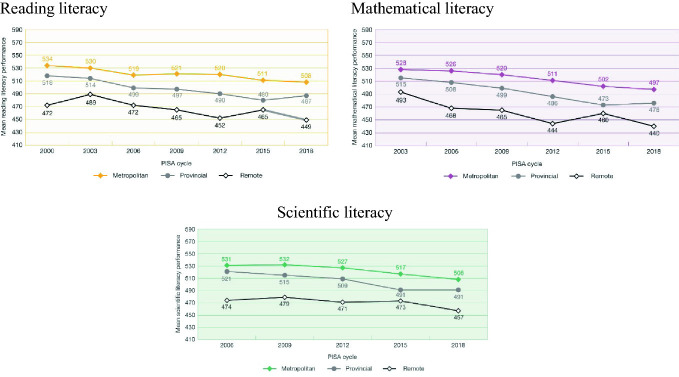
Mean reading and mathematical literacy scores over time, metropolitan, provincial and remote areas, PISA 2000–2018, Australia (*Source* Thomson et al. 2019)

The average reading literacy score for students in metropolitan schools in PISA 2018 was 508 points (Fig. 10). This achievement was significantly higher than the score for those in provincial schools of 487 points, which was in turn, significantly higher than the score for those in remote schools of 447 points. Over time, the average scores for students in both metropolitan and provincial schools have declined significantly (by 26 points and 31 points respectively), while the score for students in remote schools declined from a peak in 2003 of 489 points to the current mean of 449 points. The gap between students in metropolitan schools and those in remote schools is much the same as in 2000, and is a little less than two years of schooling.

In mathematical literacy the differences are more dramatic. The average mathematical literacy score for students in metropolitan schools in 2018 was 497 points. This achievement is significantly higher than the score for students in provincial schools of 476 points, which was in turn, significantly higher than the average score for students in remote schools of 440 points. Over time, scores declined both significantly and substantially for all groups: by 31 points for students in metropolitan schools, 39 points for those in provincial schools and 53 points for those in remote schools. The gap in performance between those in metropolitan schools and those in remote schools has gone from 35 points in 2003 (around 1.25 years of schooling) to 57 points (2 years of schooling) in 2018.

In scientific literacy in 2018 the average score for students in metropolitan schools was 508 points, 18 points higher than those in provincial schools, and 51 points higher than for those attending remote schools. Over time, scores in scientific literacy have declined by 23 points for students in metropolitan schools, 30 points for those in provincial schools and 17 points for those in remote schools. The gap in performance between students in metropolitan and remote schools has remained about the same since 2006—57 points in 2006 and 51 points in 2018. These are both around two years of schooling.

**Fig. 11:**
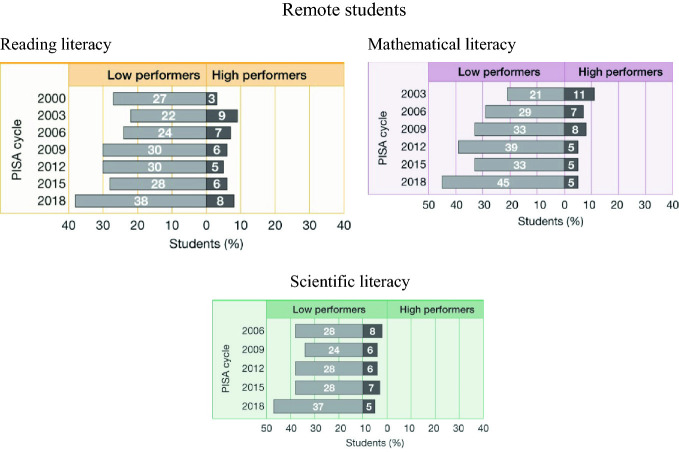
Proportions of low performers in reading, mathematical and scientific literacy for students in remote areas over time, PISA 2000–2018, Australia (*Source* Thomson et al. 2019)

In terms of proficiency levels, the proportion of low performers amongst students in rural areas has increased over time across all three assessment domains (Fig. 11). In reading literacy in 2000, 27% of rural students were low performers in 2018 this had increased to 38 per cent. In mathematical literacy the proportion has more than doubled—from 21% of students in 2003 to 45% in 2018, and in science the proportion has increased from 28% of rural students in 2006 to 37% in 2018.

## Relationship Between School Sector and Disadvantage

It is evident from the results for PISA 2018 that Australia is not meeting its own targets of excellence and equity, and it is far from being on track to meet the goal of being in the “top five by 2025” (however that goal was intended to be measured). Despite apparent increased levels of funding over the last 18 years, the introduction of a national curriculum, the establishment of national agencies to develop national standards for teaching and school leadership and a national testing program of students at a range of age levels, as well as participation in international studies of assessment, average scores have declined year after year.

In investigating the intersection of student performance and funding, it is informative to look at which schools advantaged and disadvantaged students attend, and in particular, where there are multiple layers of disadvantage.

**Fig. 12:**
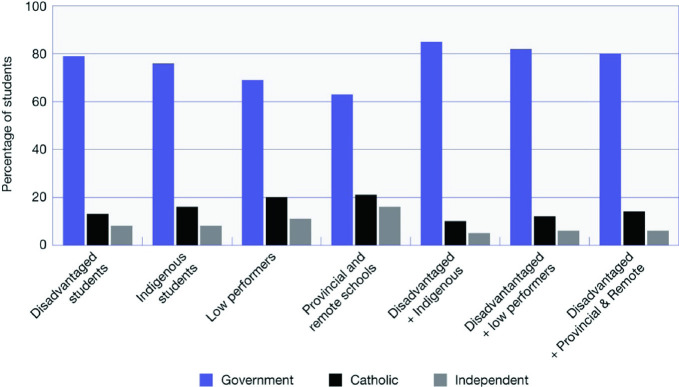
Type of school attended by disadvantaged groups, PISA 2018, Australia (*Source* OECD 2019)

Government schools enroll the vast majority of students who fall into the categories of disadvantaged groups as defined by the governments of Australia (Fig. 12). Forty-one percent of government schools can be classed as disadvantaged schools,^6^ compared to three percent of Catholic and less than one percent of independent schools. In contrast, ten percent of government schools, 31% of Catholic schools, and 63% of independent schools, and are classed as advantaged schools. Over 80% of disadvantaged students attend government schools.

Over the past 18 years, analysis of school market share using Geographic Information System (GIS) technology has found that recent enrolment shifts are largely towards government schools in high SES areas, and towards non-government schools in lower SES areas. Further analysis (Bonnor & Shepard 2016) using NAPLAN data shows that, in general, it is the more advantaged students who are moving to the more advantaged schools. As these more disadvantaged students have moved to more advantaged schools, the students remaining in schools lower down the socioeconomic scale lose diversity and talent, and their school body contains a higher proportion of disadvantaged students. This creates a cycle where some parents identify schools as low performing or high disadvantage and, if possible (that is, if they are financially able to do so), enroll their children at schools with higher proportions of advantaged students.

Table 1 provides a very brief overview of some of the differences between advantaged and disadvantaged schools from PISA 2018.^7^ These data paint a picture of less qualified and less well-prepared teachers, issues with teacher absenteeism, lack of materials and lack of physical infrastructure at a substantial proportion of disadvantaged schools, but rarely at advantaged schools.

**Table 1:** Principal’s views on hindrances to providing instruction (Australia) (*Source* OECD 2019)

		Disadvantaged schools (%)	Advantaged schools (%)
Percentage of students in schools whose principal reported that the school’s capacity to provide instruction is hindered at least to some extent by	Lack of teaching staff	34	3
	Inadequate or poorly qualified teaching staff	21	0.3
	Teacher absenteeism	28	5
	Teachers not well prepared	18	5
	Lack of educational material	21	1
	Inadequate or poor educational material	21	0.3
	Lack of physical infrastructure	45	6
	Lack of student respect for teachers	16	0.3

In addition to a lack of resources, PISA 2018 data show that 21% of students attending disadvantaged schools compared with 0.8% of students attending advantaged schools are enrolled in schools in which the principal reports that the school’s capacity for instruction is hindered at least to some extent by students intimidating or bullying other students.

## Conclusions

The factors described in this chapter have set Australia up to have a large number of young people whose experiences of education are less than they could be, and who are being failed by our current system. Many of these students cope with multiple layers of disadvantage. At present, these students are not being adequately supported by government education policies that fail to provide funding where it is most desperately needed—for basics such as infrastructure and materials, good quality teachers, or enough teachers. Their outcomes reflect this lack of provision of basic educational services. There are a substantial number of studies published in recent years which demonstrate that increased expenditure on schools improves student outcomes, particularly for disadvantaged schools and students (for example Baker 2019; Darling-Hammond 2019; Kirabo Jackson 2018).

Improving Australia’s PISA score is not an outcome in itself, it would simply be a reflection of an improvement in the health of the educational system overall, as that is what PISA was designed to measure. Improving the health of the educational system can be brought about by actively directing adequate funding to schools that are attempting to educate an increasingly diverse student population, many of whom are already experiencing multiple challenges to their engagement with and mastery of the curriculum. To a large extent these are government schools, and increased funding of these schools is essential to provide the human and physical resources needed by these students. As the OECD state:

Achieving equity in education means ensuring that students’ socio-economic status has little to do with learning outcomes. Learning should not be hindered by whether a child comes from a poor family, has an immigrant background, is raised by a single parent or has limited resources at home, such as no computer or no quiet room for studying. Successful education systems understand this and have found ways to allocate resources so as to level the playing field for students who lack the material and human resources that students in advantaged families enjoy. When more students learn, the whole system benefits. This is an important message revealed by PISA results: in countries and economies where more resources are allocated to disadvantaged schools, overall student performance in science is somewhat higher. (OECD 2016, p. 233).

**Author’s Addendum**

During the preparation of this chapter world events have not stayed still. In this time, the face of education as we know it has been forever changed, by the COVID-19 pandemic. In Australia it reached our shores in the final weeks of Term 1, 2020. Over the following 6 weeks, education systems moved at frenetic pace to bring online learning to as many students as possible, as quickly as possible. Schools were mostly closed early and required to send students some online work. It is notable that many private schools had the resources at school to provide such curricula/programming much more readily than most public schools. Government education policy had many schools for most of Term 2 and in Victoria at least, all of Term 3, positioned as places to be attended on a face-to-face basis, only by children of parents deemed as essential workers or unable to work at home, or for vulnerable students.

Such a dislocation of schooling and the planned abrupt move to online learning, and its ongoing development, brings with it added pressures in terms of the equity for all students of accessing and achieving equitable schooling outcomes. While about 87% of Australians can access the internet at home (Watt 2019), only 68% of Australian children aged 5–14 living in disadvantaged communities have internet at home, compared with 91% of students living in advantaged communities (Smith Family 2013). Of the Australian PISA students, 84% of those from disadvantaged families have access to a computer at home which they can use for study (meaning 16% do not), compared with virtually 100% of students from an advantaged background. Which would be fine for those cohorts as long as all of these families only have one student in the home. With multiple children comes the imperative for multiple computers, however only 71% of disadvantaged households, compared with 99% of advantaged households, have two or more computers. With added family stresses due to sudden working from home, unemployment of family members, associated anxiety and lack of experience or in understanding how best to facilitate children’s online learning, as well as possibly a lack of appropriate technological skills, this pandemic is almost certain to have a profound and exacerbating impact on the educational outcomes for many disadvantaged students.

Now, more than ever, Australia needs to recognize that our students do not currently benefit equally from their learning, and that online learning, especially in the context of the planned implementation, will almost inevitably worsen the achievement outcomes of the disadvantaged. To assume the nation’s students will have an equal capacity to take up and engage in online learning ‘inherently privileges the wealthy and further entrenches a multi-tiered educational model’ (Graham & Sahlberg 2020).

Australia has an opportunity here and now to make some wholesale changes to the national provision of education provision, and thus necessarily to educational funding. Policy makers and citizens have this unique opportunity, one such as they have never previously had, to insist on equitable educational provision. Future generations, with the benefit of hindsight, will clear-sightedly judge whether this opportunity was grasped with both hands or was squandered.

## Footnotes

1 Government schools are owned and operated by state or territory governments. They are almost entirely funded by taxes and nominally free for students to attend, though schools frequently charge for other expenses. Catholic schools are owned by the Catholic Church in Australia and the state Catholic education offices distribute funding and provide support to the Catholic dioceses in their state, which own and operate the schools. They receive funding from federal and state governments and charge fees. Independent schools are non-government schools that are run by a variety of private non-profit organisations, although the vast majority are governed by religious bodies. They receive funding from federal and state governments and charge fees.

2 For far more detailed accounts of this history, see Bonnor & Caro (2007), and Taylor (2018).

3 As Bonnor and Caro note, Cardinal George Pell, the then Catholic Archbishop of Sydney, commented that 43% of Catholics are educated in government schools and this figure included 69% of Catholic students from families from the lowest third of family income. “[A]s a consequence Catholic schools are not educating most of our poor … predominantly our schools now cater for the huge Australian middle-class, which they helped create” (2007, p. 109).

4 PISA surveys 15-year-old students nationally. These students are primarily found in Years 9, 10 and 11 in Australian schools. Using regression techniques an approximation can be found in each subject to the number of points that typically represent “one year of schooling” in Australian schools: 33 points on the PISA reading literacy scale, 28 points on the mathematical literacy scale, and 27 points on the scientific literacy scale.

5 The four provinces of China that participated in PISA 2018: Beijing, Shanghai, Jiangsu and Zhejiang.

6 Defined as those whose average intake of students falls in the bottom quarter of the PISA index of economic, social and cultural status within the country, compared to advantaged schools, defined as those whose average intake of students falls in the top quarter of that index.

7 Personal calculations, Australian PISA 2018 data (OECD 2019).
